# Aarskog-Scott Syndrome: A Review and Case Report

**DOI:** 10.5005/jp-journals-10005-1168

**Published:** 2012-12-05

**Authors:** Luciane Q Closs, Maximiano Tovo, Caroline Dias, Daniele P Corradi, Ivana A Vargas

**Affiliations:** Chairman, Department of Orthodontics, Lutheran University of Brazil ULBRA, RS, Brazil, e-mail: lucloss@uol.com.br; Assistant Professor, Department of Pediatric Dentistry, Lutheran University of Brazil, RS, Brazil; Graduate Student, Pedodontist, Department of Orthodontics, Lutheran University of Brazil, RS, Brazil; Department of Orthodontics, Lutheran University of Brazil, RS, Brazil; Assistant Professor, Department of Orthodontics, Lutheran University of Brazil, RS, Brazil

**Keywords:** Aarskog syndrome, Case report, Orthodontics

## Abstract

This paper reports the treatment and 12-year follow-up of a patient 7 years old who had been diagnosed with Aarskog-Scott syndrome. The patient had a history of premature multiple tooth loss, vertical dimension loss and severe dentoalveolar discrepancy. Orthopedic and orthodontic rehabilitation treatments were performed to improve the patient’s esthetic, functional and psychological condition.

**How to cite this article:** Closs LQ, Tovo M, Dias C, Corradi DP, Vargas IA. Aarskog-Scott Syndrome: A Review and Case Report. Int J Clin Pediatr Dent 2012;5(3):209-212.

## INTRODUCTION

Aarskog-Scott syndrome (ASS), also named faciogenital dysplasia, is a rare syndrome that mainly affects the musculoskeletal system of male individuals, related with X-linked and mutations in *FGD1* (Online Mendelian Inheritance in Man **#** 305400)*.* It has facial, genital and digital hands symptoms. The most common clinical findings are a round face, short stature and a bifid scrotum. Many patients also exhibit a combination of additional factors. ^1,2^

Alterations occur when individuals with this syndrome are 2 to 4 years old, with a delayed growth spurt in adolescence, rarely achieving a final height of 160 cm. Their hormone levels are normal and therefore, growth hormone treatment is ineffective. ^3^

This paper presents the 12-year follow-up of a patient with ASS and reports the dentoalveolar alterations and the treatment options that were used subsequently.

## DESCRIPTION OF CASE

A 7-year-old Caucasian boy was referred to the Department of Orthodontics and Pediatric Dentistry, School of Dentistry, ULBRA in Canoas (RS, Brazil). He had previously been diagnosed with ASS.

**Figs 1A to D:**
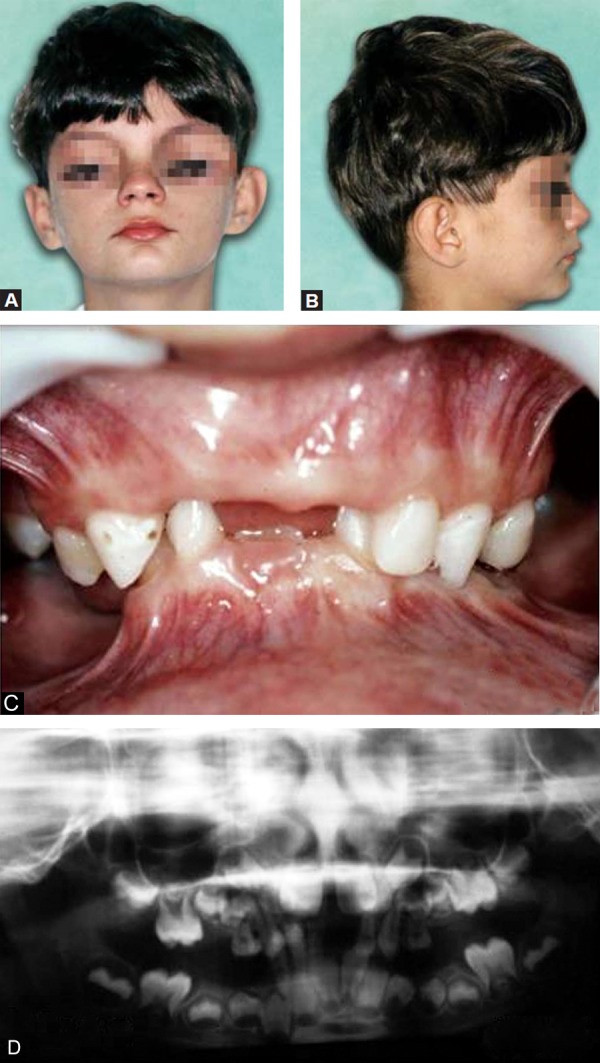
Extraoral frontal and profile view (age, 7 years)

The patient considered himself to be healthy. However, clinical characteristics of ASS were observed, such as short stature; pectus excavatum; a systolic murmur at cardiac auscultation, with features of an innocent murmur; bilateral inguinal hernias, brachydactyly; bilateral clinodactyly and a distinct interdigital membrane. Extraoral examination showed mild facial asymmetry; ocular hypertelorism, with the bipupillar midline lowered on the right side; dysmorphic ears; a straight profile; hypoplastic midface with reduced facial height; a long philtrum; thin lips; an everted lower lip and a deep mentolabial sulcus (Figs 1A and B).

Intraoral and radiographic evaluation showed the early loss of several primary teeth and some partially erupted permanent teeth. Severe loss of vertical dimension was evident, with the lower primary lateral incisors found to be in contact with the palatal region. Both the maxillary and mandibular arches showed loss of space that was accompanied by negative dentoalveolar discrepancy (Figs 1C and D).

**Figs 2A to D:**
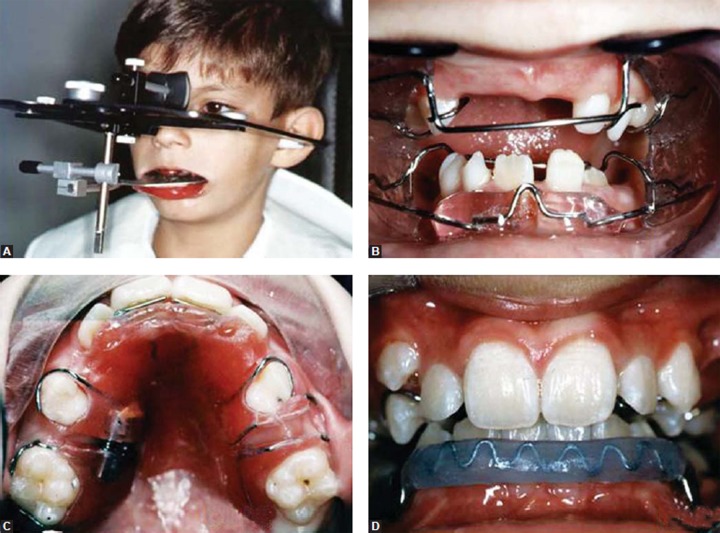
Facebow transfer for mounting models in proper vertical dimension

The Frankel II appliance was chosen for restoring the lost vertical dimension, avoiding anteroposterior and transverse collapse of the arches and supporting perioral musculature. The plaster casts were mounted on an articulator, with restoration of the vertical dimension by using a 5 mm thick posterior wax block. This appliance was used by the patient for 24 months, for a minimum of 16 hours a day (Figs 2A and B).

During the permanent tooth eruption, the following measures were used for 1 year: A removable appliance with an expansion screw was used to distalize the upper molars, and a lip bumper was used to verticalize the lower molars and to eliminate lip pressure on the lower incisors (Figs 2C and D).

In the second phase, when the patient was 12 years old, corrective orthodontic treatment was performed to allow proper eruption of all the permanent teeth and to correct a deep overbite and the class II molar relationship. A modified pendulum appliance, with an acrylic bite plane in the anterior region, was used to allow the eruption of posterior teeth for the vertical development of the arches. In the sequence, Titanium-molybdenum alloy (TMA) springs were activated to allow the distalization of the upper molars. A straight wire Roth 0.022” appliance was then used for dental alignment and leveling, along with an extraoral appliance for upper anchorage.

This treatment allowed the eruption of all the teeth and significant improvement of the deep bite, although a unilateral class II molar relationship was still observed on the left side. In a joint decision with the parents, we opted for premature interruption of treatment because the patient did not cooperate with respect to implementing the dental hygiene and was tired of a long treatment period and satisfied with the results. At this time, we used a wraparound retainer with an anterior bite plane in the upper arch and also used a bonded fixed retainer 3×3.

**Figs 3A to D:**
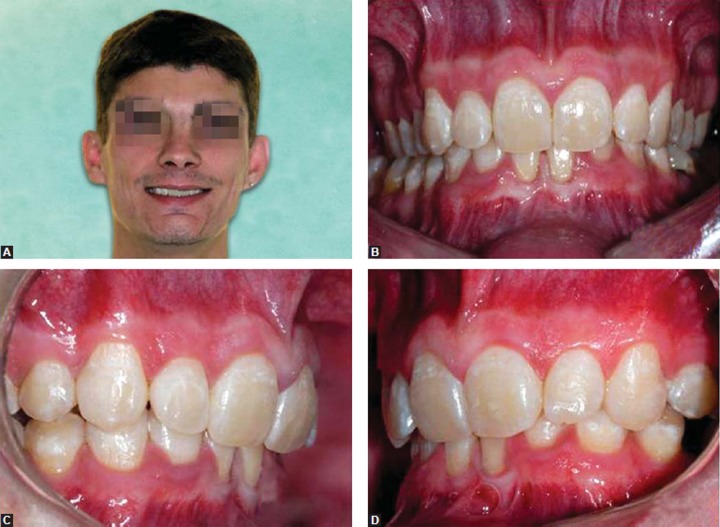
Follow-up records after 12 years of initial treatment (age—19 years) smiling and intraoral views

Five years after discontinuing orthodontic treatment, the patient returned with a deep bite relapse, and with indication for extracting first lower left premolar (severe coronary destruction due to a deep cavity). In 2010, the orthodontic treatment was resumed in order to improve the vertical relationship of the bite and to close the space between permanent mandibular left canine and permanent mandibular left second premolar (Figs 3A to D).

## DISCUSSION

In this syndrome, musculoskeletal development abnormalities in the jaws results in relative mandibular prognathism and dental crowding.^4^ Our patient had severe loss of vertical dimension, early loss of teeth and negative dentoalveolar discrepancy. Regular fluoride use, oral hygiene guidelines and dietary recommendations were implemented during treatment appointments.

The orthodontic treatment used for patients with ASS can be similar to that used for patients without the syndrome.^5^ However, in the case of our patient, the treatment lasted for a longer period than normal, probably because of the early loss of numerous teeth and the long period taken for the stimulation of the vertical development of the maxillary and mandibular arches. Patient cooperation with respect to hygiene and treatment compliance is a factor that may affect the prognosis in such cases as follow:

- What this clinical report adds – An extensive follow-up of a patient diagnosed with ASS and the clinical approach regarding interdisciplinarity pediatric dentistry- orthodontics.
- Why this case report is important to pediatric dentists – pathognomonic signs appear when individuals with this syndrome are 2 to 4 years old; they present high caries prevalence, early dental crowding (in the first transitional period of mixed dentition) and odontogenic disorders. Probably a well-informed and attentive clinician will contribute to earlier diagnosis and appropriate treatment of these patients.
